# Adult-Onset Still’s Disease Presenting as Fever of Unknown Origin: A Case Report

**DOI:** 10.7759/cureus.111957

**Published:** 2026-07-02

**Authors:** Diogo Macedo, Ana Rita de Sousa Melo, Inês Amaral Pinto, Isabel Cruz

**Affiliations:** 1 Internal Medicine, Unidade Local de Saúde de Gaia e Espinho, Vila Nova de Gaia, PRT

**Keywords:** adult-onset still’s disease, anakinra treatment, autoinflammatory disease, fever of unknown origin (fuo), hyperferritinemia

## Abstract

Adult-onset Still's disease (AOSD) is a rare systemic autoinflammatory disorder that should be considered in patients presenting with fever of unknown origin (FUO). Diagnosis is challenging due to overlapping features with infectious, autoimmune, and hematological conditions. We report the case of a 19-year-old female presenting with a six-day history of persistent fever, later developing an evanescent rash and migratory polyarthralgia. Laboratory findings included cytopenias, elevated inflammatory markers, and markedly elevated ferritin levels. After ruling out infectious, autoimmune, and neoplastic etiologies, AOSD was diagnosed based on the Yamaguchi classification criteria. Initial treatment with corticosteroids led to partial clinical improvement, but persistent articular symptoms required escalation to anakinra, resulting in complete remission. This case reinforces that AOSD should be considered in the differential diagnosis of FUO, particularly in the presence of quotidian fever, rash, polyarthralgia, and marked hyperferritinemia. Early recognition and appropriate treatment, including biological therapy, are essential to control disease activity and prevent complications.

## Introduction

Adult-onset Still’s disease (AOSD) is a rare systemic autoinflammatory disorder first described in the early 1970s. It is regarded as part of a disease continuum with systemic juvenile idiopathic arthritis (sJIA), differing primarily in the age of onset, which is defined as 16 years or older [1]. AOSD predominantly affects young adult women, and the estimated incidence ranges between 0.16 and 0.62 cases per 100,000 individuals [2].

Although the precise pathophysiological mechanisms remain unclear, AOSD is thought to result from a complex interplay between genetic susceptibility and environmental triggers. Associations with specific human leukocyte antigen (HLA) alleles have been reported, and infectious agents have been implicated as potential triggers. Stressful life events have also been proposed as contributing factors in susceptible individuals. Recent insights into innate immune dysregulation have led to the reclassification of AOSD within the spectrum of autoinflammatory disorders, driven by evidence supporting the central role of pro-inflammatory cytokines such as Interleukin‑1 (IL-1). Elevated circulating levels of these cytokines correlate with disease activity and systemic manifestations. The therapeutic efficacy of IL-1 blockade (for example, with anakinra) further supports the pivotal role of IL-1 in disease pathogenesis and reinforces the autoinflammatory framework over a purely autoimmune model [3,4].

Clinically, AOSD presents with a broad and heterogeneous spectrum of manifestations. The hallmark features include daily spiking fever, an evanescent salmon-colored rash, arthralgia or arthritis, and markedly elevated inflammatory markers, often accompanied by hyperferritinemia. However, diagnosis remains challenging due to the absence of pathognomonic biomarkers. Several classification criteria, including Yamaguchi and Fautrel criteria, have been proposed to facilitate early recognition and improve diagnostic accuracy [5-7]. This case report highlights the importance of considering AOSD in the differential diagnosis of fever of unknown origin (FUO) with systemic inflammatory symptoms, especially after ruling out infectious and autoimmune causes.

## Case presentation

A previously healthy 19-year-old woman, born in Brazil and living in Portugal for the past four years, presented to the emergency department with a six-day history of persistent fever occurring twice daily, typically in the early evening and in the afternoon, with a maximum tympanic temperature of 38.2 °C. The febrile episodes persisted despite fixed-dose antipyretic therapy consisting of alternating paracetamol and ibuprofen, without significant clinical response. The patient also reported odynophagia and a pulsatile frontal headache. She denied any other symptoms. Regarding the epidemiologic context, the patient had no recent travel except for a one-day stay in Spain, limited to an urban setting and without contact with animals or ill individuals. She denied consumption of unpasteurized dairy products or undercooked foods. Family history was remarkable for her father’s death from an unspecified type of skin cancer and a maternal aunt diagnosed with Hashimoto’s thyroiditis. Three days prior to the current presentation, the patient had been evaluated in the same emergency department by an ophthalmologist for ocular complaints and was discharged with a lubricating eye drop, with documentation of a normal ophthalmologic examination.

Upon evaluation in the emergency department, the patient was afebrile with a temperature of 37.2ºC. The oropharynx was erythematous. No skin abnormalities or palpable lymphadenopathy were noted. The remainder of the physical examination and a summary neurological examination were unremarkable. Laboratory investigations revealed mild anemia (hemoglobin: 11.4 g/dL), thrombocytopenia (107 × 10³/μL), an elevated C-reactive protein (CRP) of 23.71 mg/dL, and normal procalcitonin levels (0.22 ng/mL). Urinalysis was unremarkable. A chest X-ray showed no abnormalities, while an abdominal ultrasound revealed homogeneous splenomegaly measuring 13.1 cm in diameter. Due to the presence of a headache, a head CT scan was performed, which showed no abnormalities. Cerebrospinal fluid analysis was normal, with no evidence of infection or inflammation.

Given the presence of persistent FUO, the patient was admitted under the care of the Internal Medicine department for further evaluation. During the first eight days of hospitalization, the patient continued to experience persistent fever occurring in the early morning and occasionally in the early evening, after dinner, despite regular administration of paracetamol 1g every eight hours. Her condition further evolved with elevated transaminases (aspartate transferase (AST)/alanine transaminase (ALT): 84/113 U/L, threefold above the upper limit of normal), an erythrocyte sedimentation rate (ESR) of 38 mm/h, and a markedly elevated ferritin level of 9,642.9 ng/mL. At this stage, hemophagocytic syndrome (HPS) became a major diagnostic consideration, as the patient exhibited the typical clinical features of fever, splenomegaly, cytopenia, and markedly elevated ferritin. However, her triglyceride levels (249 mg/dL), fibrinogen (455 mg/dL), and soluble CD25 (871 U/mL) prevented the full diagnostic criteria for HPS from being met. 

**Figure 1:**
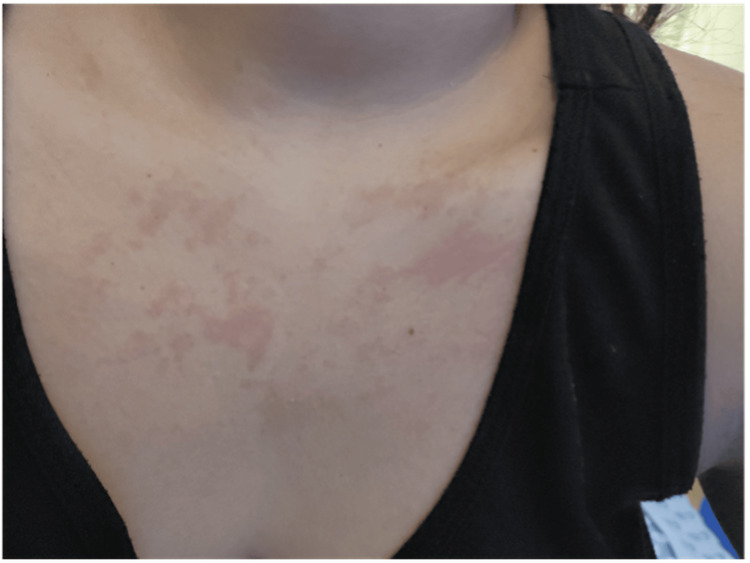
Evanescent salmon-pink maculopapular rash on the trunk during febrile episode

**Figure 2:**
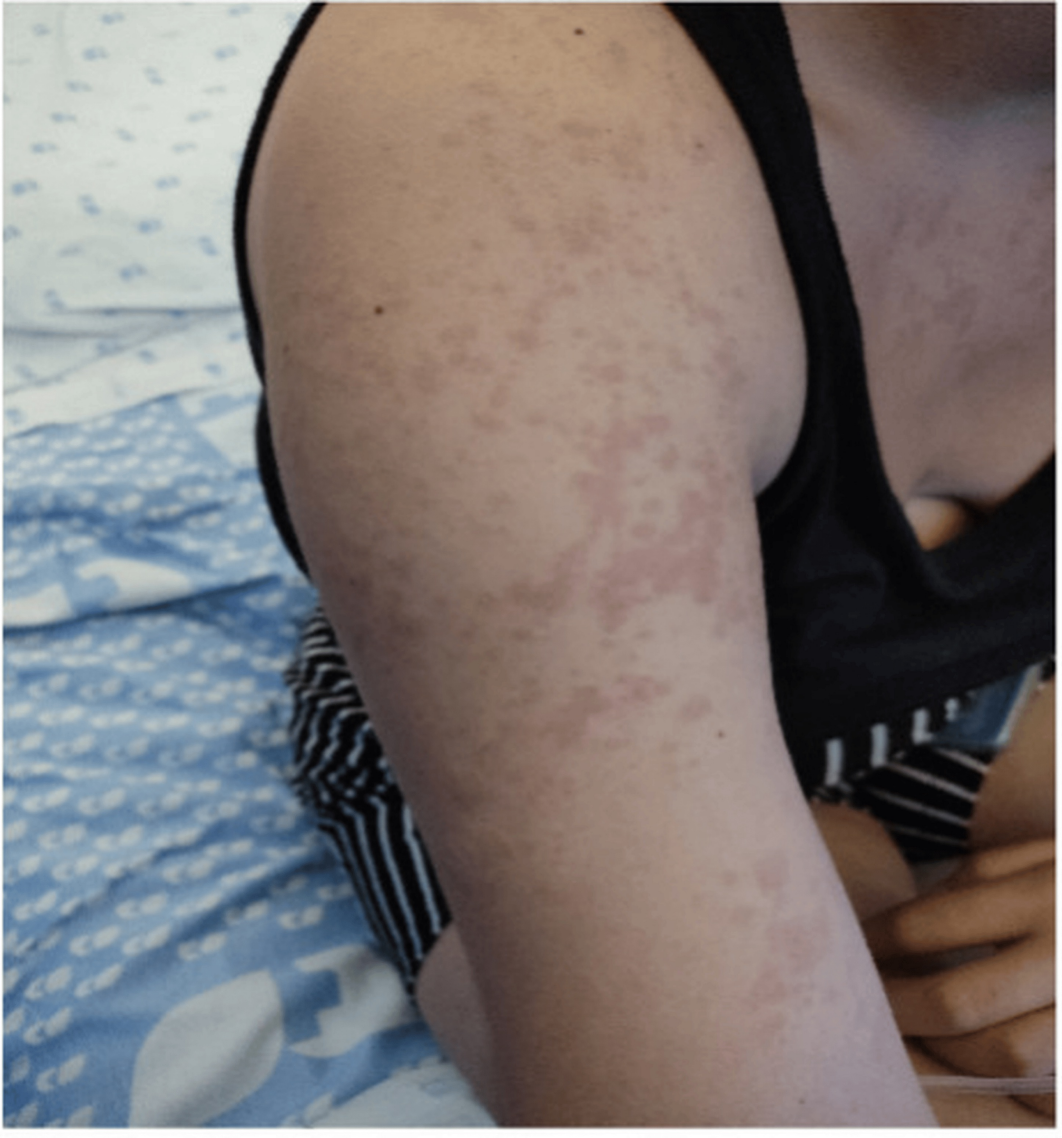
Extension of the transient rash to the right upper limb during fever peak

On the fourth day, a non-pruritic, salmon-pink maculopapular erythema appeared on the trunk and right arm during a febrile episode. The rash resolved spontaneously following defervescence, leaving no residual lesions (Figures 1, 2).

During hospitalization, the rash continued to recur with fever, additionally involving the abdomen and thighs. By day six of hospitalization, the patient developed migratory, asymmetric polyarthralgias affecting the right elbow, shoulder, and wrist, the left knee, and the third and fourth proximal interphalangeal joints on the right hand. These symptoms were more pronounced in the morning, lasted for over one hour, and improved throughout the day, suggesting an inflammatory pattern. No signs of arthritis were observed. The patient was treated with naproxen 500 mg every eight hours, providing mild relief from the articular symptoms, but the fever persisted. 

Given the clinical picture, infectious and autoimmune etiologies were investigated. Blood and urine cultures were negative. Extensive infectious workup, including blood and urine cultures and serologic testing for viral (cytomegalovirus, parvovirus B19, herpes simplex virus, human T-lymphotropic virus (HTLV) I/II, Dengue, hepatitis A, B, and C, HIV, syphilis, Epstein-Barr virus, and varicella), bacterial (*Borrelia*, *Coxiella*, *Rickettsia*) and parasitic pathogens (*Toxoplasma*, *Schistosoma*, *Echinococcus*, and *Fasciola*) was negative. Autoimmune testing revealed antinuclear antibodies (ANA) at 1:160 with a homogeneous pattern, but the remaining panel was negative, including extractable nuclear antigens (ENA), rheumatoid factor (RF), anti-cyclic citrullinated peptide (anti-CCP) antibodies, antineutrophil cytoplasmic antibodies (ANCA), and angiotensin-converting enzyme (ACE). Complement levels were normal, and serum protein electrophoresis showed no abnormalities. A total-body CT scan revealed hepatosplenomegaly without lymphadenopathy and no other abnormalities.

During hospitalization, laboratory findings evolved as follows: worsening anemia, leukocytosis, an initial thrombocytopenia that progressed to thrombocytosis, and resolution of previously elevated transaminases and alkaline phosphatase. Additionally, CRP and ESR remained significantly elevated (Table 1).

**Table 1:** Laboratory values from admission to discharge AST: aspartate aminotransferase; ALT: alanine transaminase; ALP: alkaline phosphatase; CRP: C-reactive protein; ESR: erythrocyte sedimentation rate;

Parameters	Values				Reference values
	Day 1 of hospitalisation	Day 4 of hospitalisation	Day 11 of hospitalisation	Day of discharge	
Hemoglobin	10.8 g/dL	9.6 g/dL	7.8 g/dL	9.1 g/dL	12 a 16 g/dL
Leukocytes	4.33x10^3^/uL	12.64 x10^3^/uL	17.49 x10^3^/uL	18.00 x10^3^/uL	3.80-10.60 x10^3^/uL
Platelets	110x10^3^/uL	222x10^3^/uL	660 x10^3^/uL	728 x10^3^/uL	150-440 x10^3^/uL
AST	84 U/L	44 U/L	14 U/L	11 U/L	4-33 U/L
ALT	113 U/L	57 U/L	15 U/L	12 U/L	4-50 U/L
ALP	136 U/L	119 U/L	106 U/L	94 U/L	30-120 U/L
CRP	9.65 mg/dL	7.65 mg/dL	23.98 mg/dL	10.64 mg/dL	< 0.3 mg/dL
Ferritin	---	1939 ng/mL	1179 ng/mL	700 ng/mL	12 a 306 ng/mL
ESR	>23 mm/h	---	>120 mm/h	>120 mm/h	<20 mm/h

Analyzing the patient’s clinical presentation, she met four major criteria (quotidian fever, arthralgia or arthritis, salmon-colored, non-pruritic maculopapular rash, leukocytosis with neutrophilic predominance) and three of the five minor criteria (pharyngitis, splenomegaly, abnormal liver function tests), fulfilling the Yamaguchi classification criteria for AOSD. Although ANA was positive at low titer, this finding was considered nonspecific and did not support an alternative autoimmune diagnosis. Following the diagnosis of AOSD, treatment was initiated with prednisone at 1 mg/kg/day. Within four days, the patient experienced resolution of fever and rash, along with a significant decrease in CRP and ferritin levels. However, she continued to report morning stiffness and polyarthralgia.

Given the incomplete response and the need for steroid-sparing therapy, anakinra (an IL-1 inhibitor) was introduced. Following the initiation of anakinra, the patient reported complete resolution of symptoms and was discharged on 100 mg of anakinra subcutaneously daily along with a gradually tapered dose of prednisone, with follow-up scheduled in the Internal Medicine-Autoimmune Diseases clinic.

**Figure 3:**
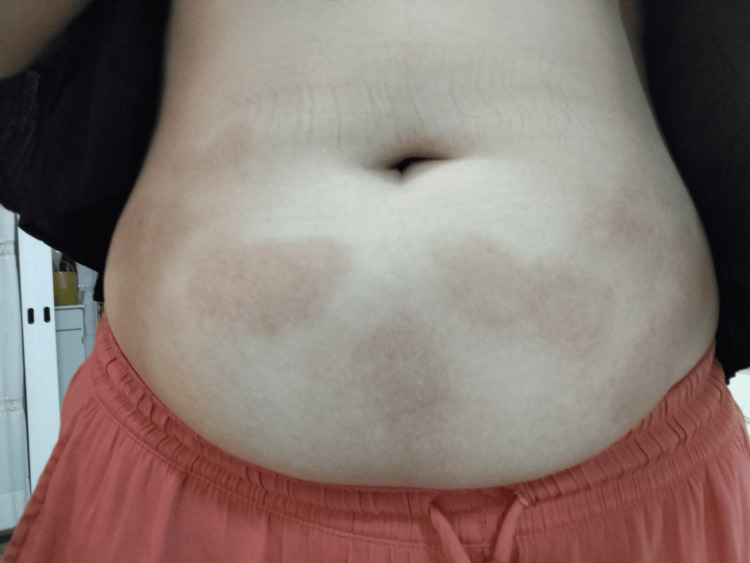
Injection-site reaction following subcutaneous anakinra administration

**Figure 4:**
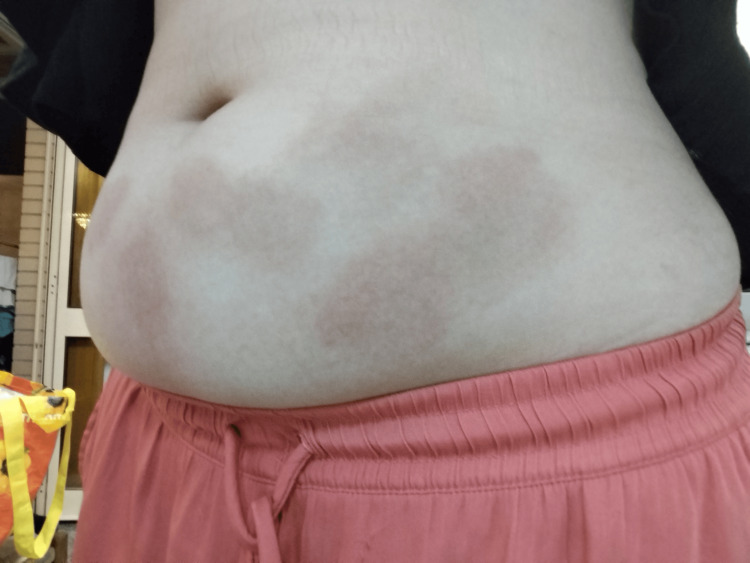
Localized erythematous reaction at anakinra injection site

One month after discharge, the patient remained asymptomatic while on anakinra and prednisone (10 mg per day). She initially developed localized cutaneous reactions at the injection site, which did not improve with topical corticosteroids or antihistamines (Figures 3, 4), but eventually resolved within two months. Corticotherapy was subsequently tapered until suspension 10 weeks after the diagnosis, with maintained clinically inactive disease. 

Six months after corticoid interruption, and due to sustained disease remission, the patient started tapering anakinra, reducing one injection per week every two to three months, with the goal of complete suspension.

## Discussion

This case highlights the diagnostic challenge posed by AOSD in patients presenting with fever of unknown origin, particularly when hyperferritinemia and cytopenias raise concern for hemophagocytic syndromes. AOSD remains a diagnosis of exclusion, and a systematic evaluation to rule out infectious, autoimmune, and malignant conditions is essential.

FUO encompasses a broad differential diagnosis. In this patient, extensive microbiological and serological investigations excluded viral, bacterial, and parasitic infections. Autoimmune diseases, including systemic lupus erythematosus, rheumatoid arthritis, and systemic vasculitides, were considered unlikely based on negative disease-specific autoantibodies, normal complement levels, and absence of compatible clinical features. Hematologic malignancy, particularly lymphoma, was also a key consideration given the presence of persistent fever and hepatosplenomegaly; however, imaging studies showed no lymphadenopathy, and laboratory findings did not support a lymphoproliferative disorder. 

AOSD manifests in three distinct patterns: monocyclic, polycyclic, and chronic articular [8]. The monocyclic pattern consists of a single episode that resolves within months, whereas the polycyclic form is characterized by recurrent flares interspersed with periods of remission lasting years. The chronic articular pattern is associated with persistent polyarthritis, often resembling other forms of inflammatory arthritis. Clinically, it is characterized by a heterogeneous presentation, typically including quotidian spiking fever, evanescent salmon-colored rash, inflammatory arthralgia or arthritis, pharyngitis, hepatosplenomegaly, and elevated acute-phase reactants. Fever is the most prominent symptom occurring in 46-100% of the patients, usually being the first sign, and sometimes being intermittent. Transient non-pruritic, salmon colored rash during febrile episodes is also typical and appears mainly on the trunk. Arthralgias are a common manifestation of AOSD and may range from mild monoarticular symptoms to severe polyarticular involvement, most frequently affecting the knees, elbows, shoulders, and proximal interphalangeal joints while often sparing the distal joints. The migratory pattern of arthralgia may mimic other inflammatory conditions, including viral arthritis, acute rheumatic fever, and disseminated gonococcal infection [8]. However, the constellation of persistent quotidian fever, odynophagia, transient salmon-colored rash temporally associated with febrile peaks, polyarthralgia with inflammatory characteristics, hepatosplenomegaly, leukocytosis with neutrophilic predominance, elevated inflammatory markers, liver enzyme abnormalities, and marked hyperferritinemia strongly supported the diagnosis in this case.

Markedly elevated ferritin levels are a well-recognized laboratory hallmark of AOSD and often correlate with disease activity. However, extreme hyperferritinemia also raises suspicion for hemophagocytic lymphohistiocytosis (HLH), a life-threatening hyperinflammatory syndrome that shares overlapping clinical and laboratory features with severe AOSD. In the present case, ferritin levels exceeded 9,000 ng/mL and were associated with cytopenia and hepatosplenomegaly, warranting careful evaluation for HLH. Nevertheless, the patient did not fulfill the HLH-2004 classification criteria [9], as triglyceride and fibrinogen levels were not compatible with HLH, soluble CD25 levels were not significantly elevated, and there was no evidence of progressive organ dysfunction. Distinguishing between AOSD and HLH is critical, as HLH requires urgent, aggressive immunosuppressive therapy, whereas AOSD may respond to corticosteroids and targeted biologic agents [10].

The diagnosis of AOSD was supported by fulfillment of the Yamaguchi classification criteria, which remain the most widely used and validated criteria for this condition. This patient met four major criteria (fever ≥1 week, arthralgia ≥2 weeks, typical rash, and leukocytosis with neutrophilia) and three minor criteria (pharyngitis, splenomegaly, and abnormal liver function tests), in addition to exclusion of alternative diagnoses [6]. Although negative ANA and rheumatoid factor are included among the original Yamaguchi exclusion criteria, low-titer ANA positivity has been reported in a minority of patients with AOSD and, in the absence of clinical or serological evidence of another connective tissue disease, should not preclude the diagnosis. In our patient, ANA positivity was considered a nonspecific finding because the remaining autoimmune investigations were negative, and no clinical features supported an alternative autoimmune disease.

The Yamaguchi classification criteria for AOSD comprise three categories: (1) Major criteria, which include (i) fever lasting for one week, (ii) arthralgia or arthritis for at least two weeks, (iii) typical nonpruritic salmon-pink skin rash, and (iv) leukocytosis of at least 10,000/mm^3^ with >80% polymorphonuclear cells; (2) Minor criteria, which include (i) sore throat, (ii) lymph node enlargement, (iii) hepatomegaly or splenomegaly, (iv) abnormal liver function tests, and (v) negative ANA and RF tests; (3) Exclusion criteria, which include (i) infections (especially sepsis and infectious mononucleosis), (ii) malignancy (mainly malignant lymphoma), and (iii) other rheumatic disorders [6]. For diagnosis of AOSD, the patient should meet five or more criteria, of which at least two should be major.

Early recognition and prompt initiation of therapy are essential in AOSD to control systemic inflammation and prevent complications. The most severe complication is macrophage activation syndrome (MAS), a secondary form of HLH associated with significant mortality. Clinically, MAS should be suspected in the presence of a change from the typical quotidian fever pattern to persistent fever, worsening cytopenias, rapidly rising ferritin levels, hypertriglyceridemia, hypofibrinogenemia, liver dysfunction, or emerging organ failure. In this case, although HLH/MAS was initially suspected, the absence of progressive laboratory deterioration and organ dysfunction supported the diagnosis of uncomplicated systemic AOSD [8,10]. 

Therapeutic strategies in AOSD aim to suppress cytokine-driven inflammation. Nonsteroidal anti-inflammatory drugs can be used to reduce fever and arthralgia during diagnosis workup, but have no role in AOSD. Systemic corticosteroids remain the mainstay of initial therapy. In this patient, high-dose prednisone (1 mg/kg/day) led to the rapid resolution of fever and rash; however, inflammatory arthralgia persisted. This clinical course is consistent with previous studies demonstrating that glucocorticoids are generally more effective in controlling systemic features than articular manifestations [8]. The persistence of joint symptoms, together with the need to minimize prolonged corticosteroid exposure, supported early escalation to targeted biologic therapy. Initiation of anakinra led to complete resolution of articular complaints, highlighting the central role of IL-1-mediated inflammation in both systemic and musculoskeletal disease activity.

The 2024 European Alliance of Associations for Rheumatology (EULAR) recommendations advocate early introduction of biological therapy targeting IL-1 or IL-6 pathways, with the aim of achieving rapid disease control and minimizing prolonged corticosteroid exposure [11]. Anakinra, an IL-1 receptor antagonist, has demonstrated efficacy in both systemic and chronic articular phenotypes of AOSD, reducing inflammation and preventing progression to joint destruction, and is associated with a favorable safety profile. In this case, initiation of anakinra resulted in complete clinical remission and allowed progressive tapering of corticosteroids. Injection-site reactions occurred but were self-limited.

The long-term prognosis of AOSD depends on disease phenotype. Patients with predominantly systemic involvement may achieve sustained remission, whereas those evolving to chronic articular disease may require prolonged immunomodulatory therapy to prevent joint damage. Close follow-up is essential to monitor for relapse and potential complications, including MAS and secondary amyloidosis. At one-year follow-up, our patient remained asymptomatic under IL-1 inhibition tapering, suggesting favorable early disease control.

## Conclusions

This case underscores the importance of considering AOSD in the differential diagnosis of FUO accompanied by systemic inflammation and hyperferritinemia. Careful exclusion of infectious, malignant, and autoimmune conditions, alongside the structured application of classification criteria, is essential for timely diagnosis. Early initiation of targeted biologic therapy plays a pivotal role in achieving disease control while minimizing corticosteroid-related toxicity.
